# Differential Effect of Culture Temperature and Specific Growth Rate on CHO Cell Behavior in Chemostat Culture

**DOI:** 10.1371/journal.pone.0093865

**Published:** 2014-04-03

**Authors:** Mauricio Vergara, Silvana Becerra, Julio Berrios, Nelson Osses, Juan Reyes, María Rodríguez-Moyá, Ramon Gonzalez, Claudia Altamirano

**Affiliations:** 1 School of Biochemical Engineering, Pontificia Universidad Católica de Valparaiíso, Valparaíso, Chile; 2 CREAS CONICYT-REGIONAL, GORE Valparaíso, Valparaíso, Chile; 3 Institute of Chemistry, Pontificia Universidad Católica de Valparaiíso, Valparaíso, Chile; 4 Department of Chemical and Biomolecular Engineering, Rice University, Houston, Texas, United States of America; 5 Department of Bioengineering, Rice University, Houston, Texas, United States of America; Universite Libre de Bruxelles (ULB), Belgium

## Abstract

Mild hypothermia condition in mammalian cell culture technology has been one of the main focuses of research for the development of breeding strategies to maximize productivity of these production systems. Despite the large number of studies that show positive effects of mild hypothermia on specific productivity of r-proteins, no experimental approach has addressed the indirect effect of lower temperatures on specific cell growth rate, nor how this condition possibly affects less specific productivity of r-proteins. To separately analyze the effects of mild hypothermia and specific growth rate on CHO cell metabolism and recombinant human tissue plasminogen activator productivity as a model system, high dilution rate (0.017 h^−1^) and low dilution rate (0.012 h^−1^) at two cultivation temperatures (37 and 33°C) were evaluated using chemostat culture. The results showed a positive effect on the specific productivity of r-protein with decreasing specific growth rate at 33°C. Differential effect was achieved by mild hypothermia on the specific productivity of r-protein, contrary to the evidence reported in batch culture. Interestingly, reduction of metabolism could not be associated with a decrease in culture temperature, but rather with a decrease in specific growth rate.

## Introduction

The market of biopharmaceutical products and drugs based on recombinant proteins (r-proteins) is growing rapidly, with total sales reaching more than 138 billion dollars in 2010 [1]. The mammalian cell culture system is predominant in the synthesis of biopharmaceutical products due to their ability to properly develop processes for assembly, folding and post-translational modifications, such as glycosylation [2].

While in the past 20 years production systems have improved in terms of cell growth, from maximum cell concentrations of 1–2 to 10–15 million cells/ml, as well as in specific (10–20 to 50–90 pg/cell/day) and volumetric (0.05–0.1 to 1–5 g/L) productivities [3], the demand for this growing market still requires further production capacity under stringent optimization schemes [4]. In this aspect, considering that the productivity of r-proteins is directly proportional to the mass of viable cells, culture viability and longevity, different approaches have been investigated to optimize the production capacity of the cultures. One of such approaches has been the use of mild hypothermia condition with temperatures ranging between 30°C and 33°C, which have been shown to increase culture longevity and specific productivity of a wide range of recombinant proteins, in addition to reducing cell growth rate in CHO cells batch cultures [5], [6], [7], [8], [9], [10], [11], [12], [13], [14].

The mechanism for an increase in specific productivity of recombinant proteins from a decrease in cultivation temperature in batch cultures has not been determined with certainty [15]. Among the possible causes that could be involved in this phenomenon are: cell cycle arrest in G1 phase, considered as a more metabolically active phase [16], [8], [17], [18], [13]; reduced or delayed catabolism of carbon and energy sources [19], [20]; increased levels of transcription and increased mRNA stability of r-proteins [21], [8], [10], [22]; increase in folding capacity and expression of endoplasmic reticulum chaperones [23], [24], [25].

While the benefits of mild hypothermia condition have been widely demonstrated by cold shock [5], [13], [20] or after adaptation of the cells [14]; [26], a significant increase in specific productivity of r-proteins has been observed in most cases, all existing reports have used batch cultivation [27], [7], [19], [10], [28], [9], [29], [30], [31], [14]. Unfortunately, this mode of cultivation does not allow to study the effect of reduced culture temperatures on r-protein productivity separately, since a reduction of the specific growth rate is simultaneously caused by mild hypothermia. In this sense, chemostat culture resurgence as the most appropriate tool for obtaining biologically reliable and homogeneous data [32], [33], [34], [35] (based on the advantages offered through environmental control, reproducibility and delivering constant physicochemical conditions), allowing control the specific growth rate of the cells (μ), through the dilution rate (D), after reaching steady state (SS) being valid in mammalian cell technology to high viability (D = μ). Thus, chemostat culture emerges as a relevant option to achieve a correct understanding of the cell behavior to solve the problem of evaluating various culture variables [36].

Therefore, the use of chemostat cultures is a more suitable option to study the sole effect of temperature changes, as it allows defining the specific growth rate by setting the dilution rate (D = F/V), (F: Feed flow; V: Reaction volume). This constant and stable environment, in turn, allows separating the specific growth rate from other system variables, therefore maintaining the cell population in a defined physiological state [37].

This study aims to separately assess the effect of mild hypothermia and specific growth rate on the production of recombinant human tissue plasminogen activation (rht-PA) produced by CHO cells. For this purpose, chemostat cultures were carried out at two D (0.017 and 0.012 h^−1^) and two culture temperatures (37°C and 33°C).

## Materials and Methods

### Cell line and culture medium

The ht-PA producing cell line (CHO TF 70R) was obtained from Pharmacia & Upjohn S.A. (Sweden) (kind gift of Torsten Björlig). The culture medium was SFM4CHO HyClone, free of glucose and glutamine, supplemented with 10 mM glucose (G7021, Sigma, USA) and 6 mM glutamate (G8415, Sigma, USA) used in substitution of glutamine [38] in the feed.

### Chemostat cultures

Chemostat culture experiments were performed in a Biostat A Plus bioreactor (Sartorius Stedim Biotech S.A., France) maintaining a working volume of 500 ml and a pH level of 6.9 (Hepes buffer, Sigma-Aldrich). The bioreactor was inoculated and operated in batch-mode during 48 h and it was then supplied with sterile feed throughout the period of operation.

A series of four experiments was performed, in duplicate, at 37°C or 33°C, keeping a High-D: 0.017 h^−1^ (Residence time (τ = 1/D), τ_0.017_: 58.8 h) or Low-D: 0.012 h^−1^.(τ_0.012_: 83.3 h). Samples were taken every 24 h for viable cell quantification, centrifuged and the supernatant was immediately frozen at −20°C for analytic measurements. Cultures were considered to reach steady-state (SS) when, after at least four residence times, 4×τ_0.017_: 235.2 h or 4×τ_0.012_: 333.2 h, and the number of viable cells, glucose, and lactate concentration, were constant in two consecutive samples [33].

### Analytical methods

Cells were counted using a hemacytometer (Neubauer, Germany). Cell viability was determined by the method of exclusion using trypan blue (T8154, Sigma, USA) (1∶1 mixture of 0.2% trypan blue in saline and cell sample). Glucose and lactate concentrations were determined with an automatic biochemistry analyzer (YSI 2700, Yellow Springs Inc., USA). The concentration of rht-PA was quantified by enzyme immunoassay (Biopool Imulyse t-PA kit, Diagnostic International, Germany).

### Quantitative real-time PCR assay

RNA extraction and cDNA synthesis were performed as previously described [39]. Cells were harvested by centrifugation at 500 g for 10 min at 4°C and stored at −80°C with RNAlate solution (RNA stabilization and protection solution). Total RNA was isolated and purified from the samples using the High Pure RNA Isolation kit (Roche Applied Science), and the concentration of RNA was determined by measuring the ratio of absorbance at 260 and 280 nm. The synthesis of cDNA was carried out using RevertAi H First Strand cDNA Synthesis kit (Fermentas Inc.) using random DNA primers according to the manufacturer's protocol. Quantitative real-time PCR (qPCR) was performed using LightCycler FastStart DNA Master SYBR Green I systems (Roche Applied Science). The sequences of the primers used for cDNA synthesis and for qPCR were designed using algorithm Primer 3 (Table 1). The level of *cdc42* mRNA was used as an internal control [40] to normalize the results obtained for the *rht-PA* mRNA. The relative quantification of gene expression was performed as described previously [39] using the standard curve method with three measurements for each gene for each experimental condition evaluated. A maximum standard deviation (SD) of 10% was obtained.

**Table 1:** Primers used in qPCR analysis.

Gene	Fordward (5′ → 3′)	Reverse (5′ → 3′)
*cdc42*	CCTCACACAGAAAGGCCTAAA	TGGGGTTCTGTGCTGTGTAA
*rht-PA*	GCCCTGGTGCTACGTCTTTA	AACACCAGCTGTGCAGAAAC

### Estimation of specific rates at Steady-State

Specific growth rate (μ) was calculated from a mass balance in the reactor: 
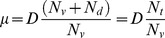
(Ec1)where N_v_ is the concentration of viable cells (10^6^/ml), N_d_ is the concentration of dead cells (10^6^/ml), N_t_ is the concentration of total cells, and D is the dilution rate of the culture (h^−1^).

Specific rates of production or consumption (Ec2.) of metabolite i (q_i_) were calculated from a mass balance in the reactor: 
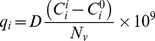
(Ec2)where C^i^_i_ is the concentration of i in the inlet (mmol/L), C^i^_o_ is the concentration of i in the outlet (mmol/L), N_v_ is the concentration of viable cells (10^6^/ml), and D is the dilution rate of the culture (h^−1^).

### Statistical Analysis

Chemostat cultures at each condition were performed in duplicate and two independent samples were taken at each time point for every culture with analytical measurements carried out separately. Values are expressed as mean standard error. Analysis of variance for factorial design of two factors was used to compare the results by Design-Expert 7 for Windows.

## Results and Discussion

### Effect of mild hypothermia and specific growth rate on cell growth

**Figure 1:**
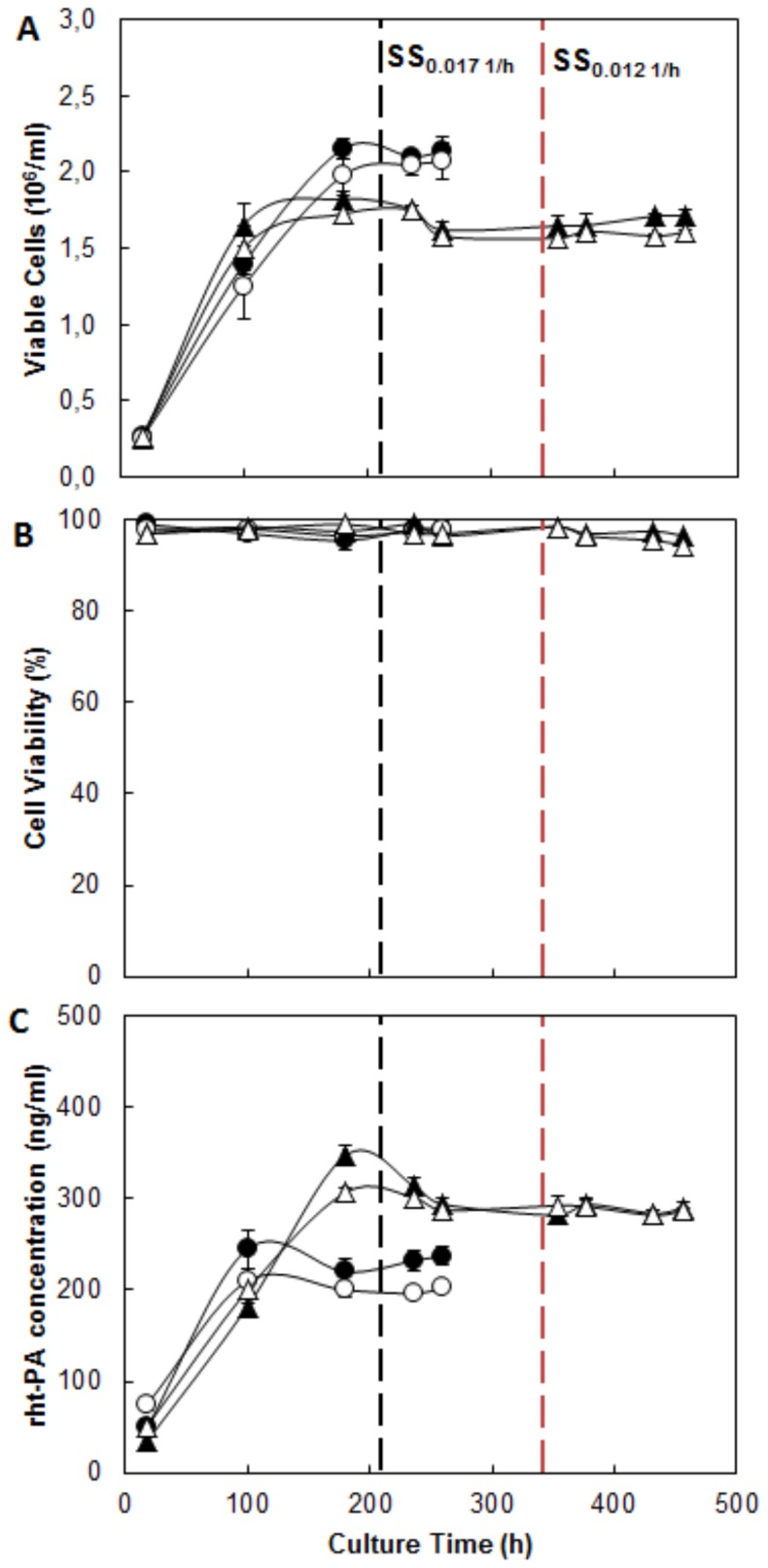
Profile of CHO cells growing, viability and rht-PA production at different culture temperatures and dilution rates. A: Viable cell concentration. B: Viability percentage. C: rht-PA concentration. • Condition 37°C and 0.017 h^−1^; ○ Condition 33°C and 0.017 h^−1^; ▴ Condition 37°C and 0.012 h^−1^; ▵ Condition 33°C and 0.012 h^−1^. Black dotted line: Start of SS 0.017 h^−1^. Red dotted line: Start of SS 0.012 h^−1^.

Separate temperature reduction and specific growth rate effects were investigated in chemostat culture, once a steady state was reached (after 4 residence times). The main results are presented in Figure 1. The maximum cell density achieved in the cultures grown at High-D (D = 0.017 h^−1^) was 2×10^6^ cells/ml±0.1, without any observed effects on temperature. In cultures grown at Low-D (D = 0.012 h^−1^) cell concentration was slightly lower, reaching a peak of 1.8×10^6^ cells/ml±0.2 at 37°C and 1.6×10^6^ cells/ml±0.1 at 33°C. Thus, a reduction by 20% is observed in cell density between the cultures carried out at 33°C. Regarding the cell viability (see Figure 1b) just at 33°C and Low-D, a slight decrease was observed, keeping all the other cultures at 97%.

These results are consistent with those reported in literature, where at the same dilution rate ranges, it has been observed a steady concentration of viable cells, both CHO [41] as hybridoma cells [42] at 37°C as cultivation temperature. However, the observed behavior at 33°C, where a decrease in the number of viable cells is observed, has been reported only in chemostat culture at 37°C and low dilution rate, close to 0.010 h^−1^ [41], [43], [44], associated with a higher rate of cell death in the G1 phase of the cell cycle, which predominate at low dilution rates [45], [46].

To date, no other studies employing chemostat culture have evaluated the effect of temperature on the number of viable cells. The response observed at High-D contradicts the idea that the hypothermia condition by itself affects cell concentration in batch cultures [8], [19], [10], [14], since no change in a viable cell number could be observed at High-D, due to different culture temperature.

### Effect of mild hypothermia and specific growth rate on production of rht-PA

**Figure 2:**
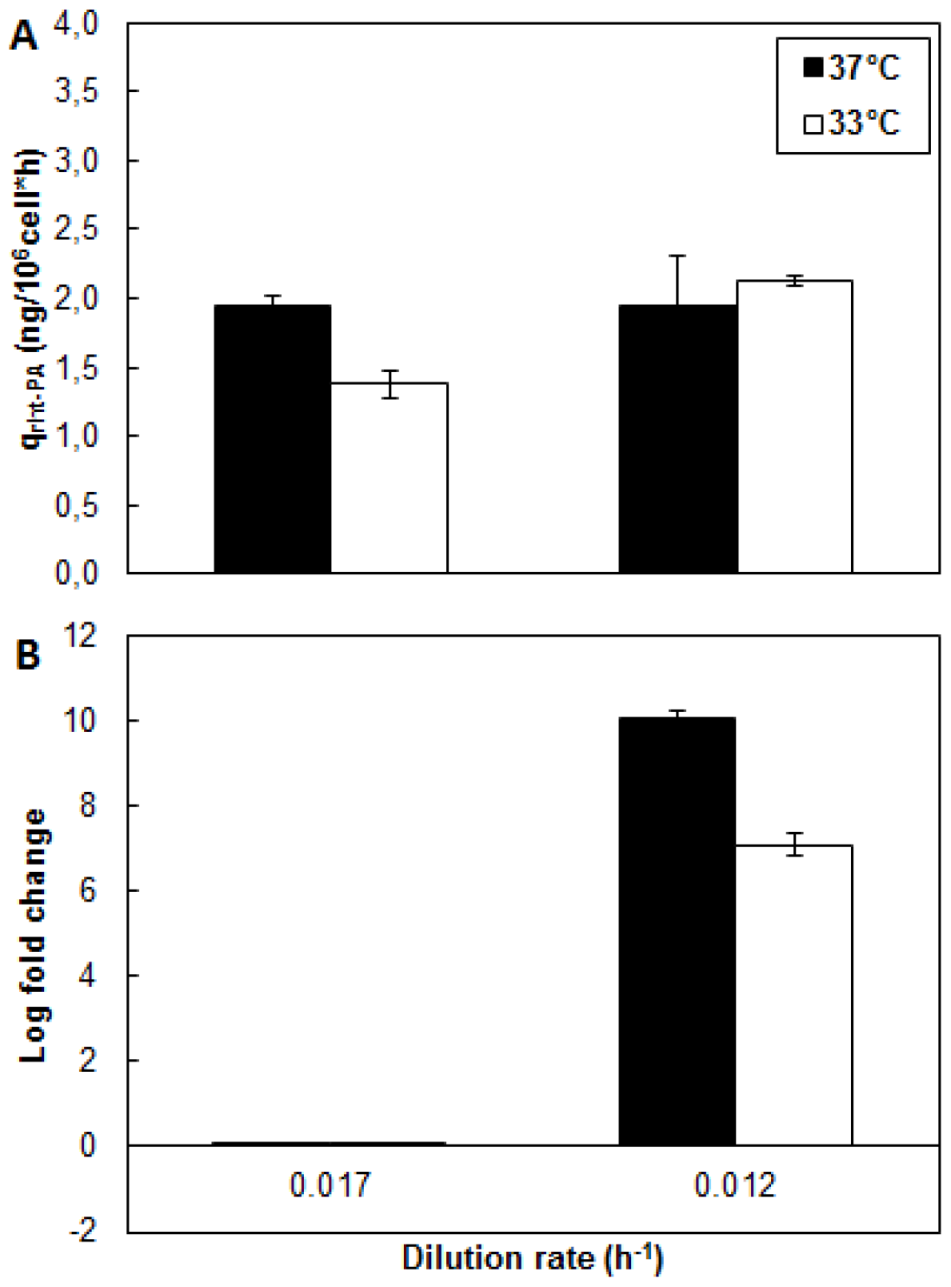
Differential effect of culture temperature and dilution rate on protein production and mRNA expression of rht-PA. A: Specific productivity of rht-PA. B: Log fold change of mRNA of rht-PA. ▪ Condition 37°C; □ Condition 33°C.

rht-PA protein production was evaluated under High-D and Low-D at two culture temperatures (37 and 33°C) (Figure 1c). Interestingly, reducing dilution rate at 37°C not produce differences in q_tPA_ (Figure 2). However, at 33°C an increase of 65% in qtPA was observed by reducing the dilution rate. Meanwhile, the temperature reduction at High-D promoted a decrease in qtPA, whereas at Low-D there were no significant differences in qtPA at both temperatures tested. These results indicate that at low specific growth rates, the use of mild hypothermia conditions influence the productivity of the recombinant protein positively. In this study the condition of Low-D could represent a turning point in relation to the effect of temperature and it requires studies at even lower dilution rates to corroborate this hypothesis. On the other hand, at Low-D levels of mRNA of rht-PA show a significant increase for both temperatures evaluated (Figure 2b). This increase in the level of mRNA only resulted in an increase in q_rht-PA_ at 33°C, without observing any increase in this parameter at 37°C.

In concordance with reported by Kou and colleagues (2011) [47], it may be presumed that post-translational processes are improved in batch culture of CHO cells, under mild hypothermia and low specific growth rate, allowing an increase in the synthesis of the rht-PA protein.

This results regarding the effect of μ on the productivity of r-protein at 33°C differ from those reported in chemostat cultures of CHO cell at 37°C [41], in which it was reported that a higher specific growth rate is associated with an increased specific productivity of r-protein. However, it's agree with that reported by Berrios et al. (2009) who shows a correlation between increasing q_rht-PA_ and decreasing μ promoted by low temperature or varying concentrations of mannose in batch culture. The relationship between specific growth rate and specific productivity is suggested to be dependent on the cell line used, the r-protein studied, as seen in the case of hybridoma culture [43], [44], [42], the limiting nutrient of culture, and also depend on culture temperature used.

Furthermore, the null of mild hypothermia effect on q_rht-PA_ in chemostat cultures at Low-D is opposite to the widely reported positive effect in batch cultures [6], [8], [19], [12], [20], [27], [9], [48], [30], [14], [31]. Meanwhile, in the condition of High-D a negative effect of mild hypothermia on q_rht-PA_ is observed. While these results have no precedents to be contrasted, at less in chemostat culture, overall, they suggest that the reported effect of mild hypothermia promoting specific productivity of r-proteins in batch CHO cell cultures, not only corresponds to the result of lowering the culture temperature, but rather is the combined effect of a temperature reduction and a reduction of cellular growth rate. Some reports suggest that a number of key regulatory proteins and pathways are involved in modulating the response of cells to mild hypothermia, such as cold shock proteins, but these were no the objective of this work.

### Effect of mild hypothermia and specific growth rate on Central Metabolism

**Figure 3:**
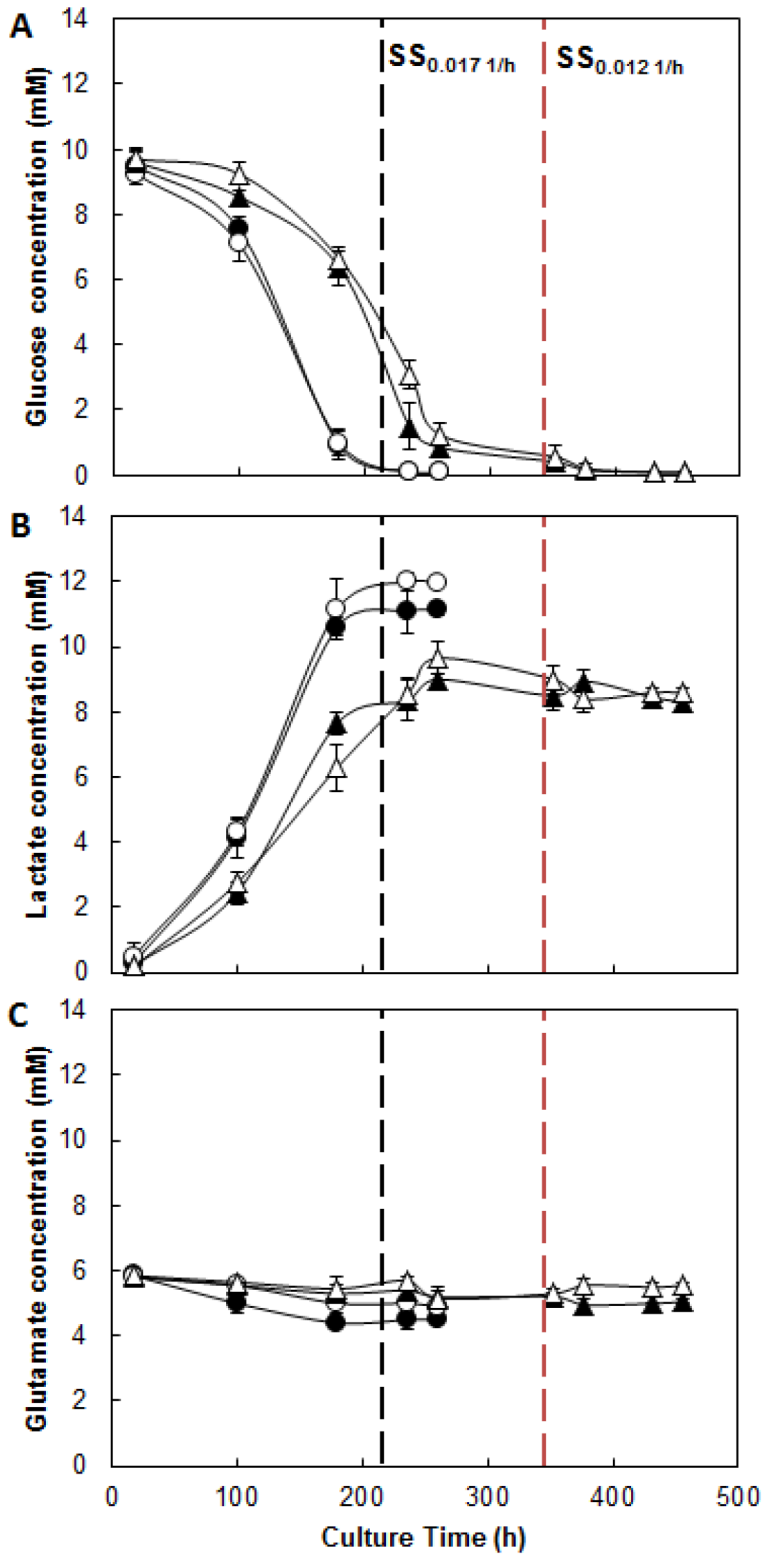
Metabolic profile of CHO cells growing at different culture temperatures and dilution rates. A: Glucose concentration. B: Lactate concentration. C: Glutamate concentration. • Condition 37°C and 0.017 h^−1^; ○ Condition 33°C and 0.017 h^−1^; ▴ Condition 37°C and 0.012 h^−1^; ▵ Condition 33°C and 0.012 h^−1^. Black dotted line: Start of SS 0.017 h^−1^. Red dotted line: Start of SS 0.012 h^−1^.

**Figure 4:**
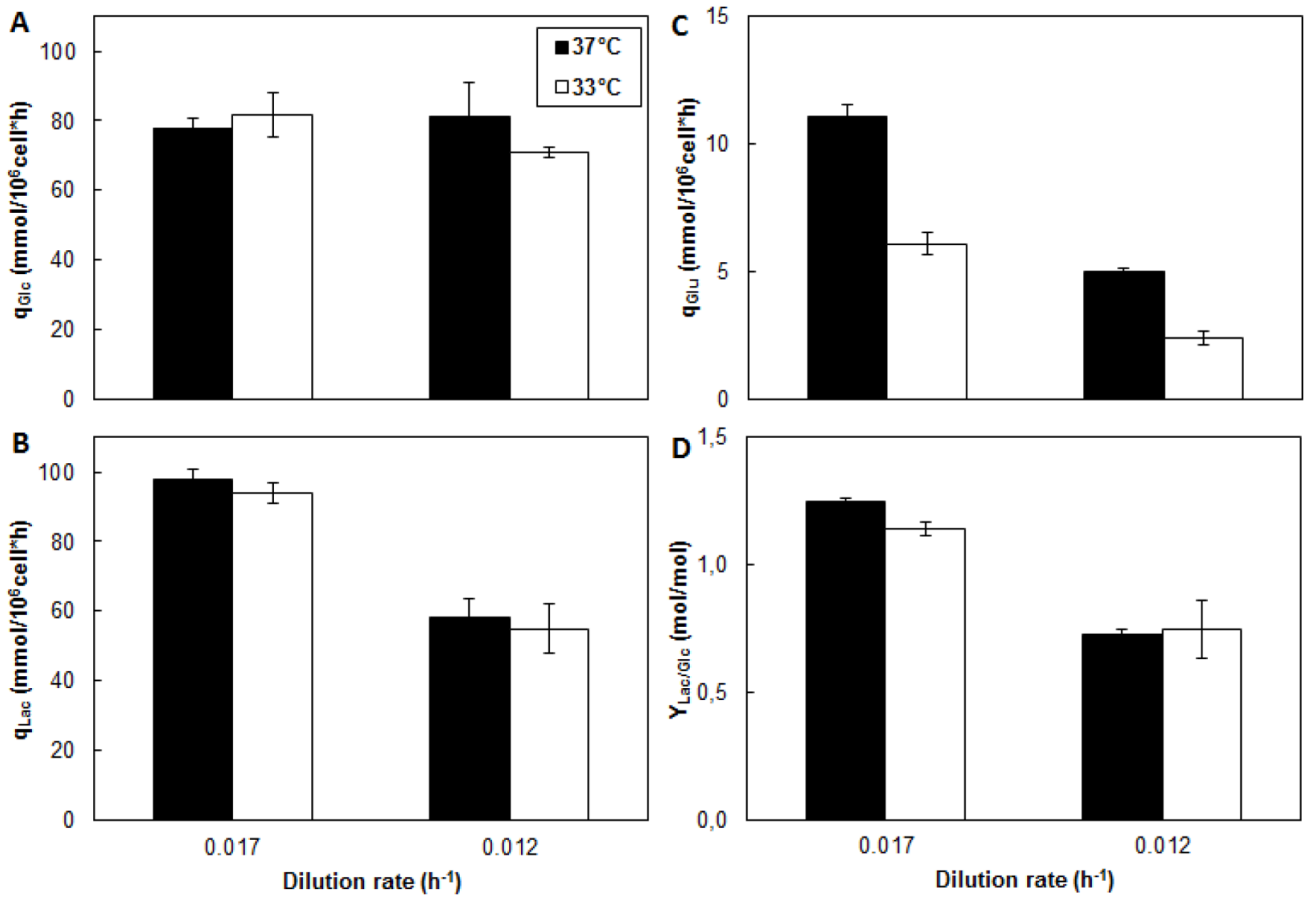
Metabolic behavior of CHO cells growing at different culture temperatures and dilution rates. A: Specific rate of glucose consumption. B: Specific rate of lactate consumption. C: Specific rate of glutamate consumption. D: Lactate yield from glucose. ▪ Condition 37°C; □ Condition 33°C.

Glucose, lactate and glutamate metabolism was investigated under two different cultivation temperatures and High-D and Low-D. The Glucose profile (Figure 3a) shows insignificant differences in the levels of residual glucose in the bioreactor, due to the reduction of culture temperature or dilution rate, in all cases, concentrations were less than 0.2 mM. Similarly, the specific glucose consumption was not affected by the temperature reduction (Figure 4a).

The use of High-D or Low-D caused no effect on the specific glucose consumption. This response differs from that reported in the literature, where a reduction in dilution rate is accompanied by a significant decrease in residual glucose levels and specific glucose consumption in CHO [41] and other cell lines [44], [42], [32]. The response obtained in this study could be explained by a redistribution of carbon fluxes for biomass and the lactate production [38]. Indeed, although the supplied glucose is capable of supporting the growth of cells and the production of recombinant protein due to the growth conditions, it is fully used and the effect of varying the dilution rate is reflected in the decreased specific lactate production. (see below).

The absence of an effect from mild hypothermia is also in contrast with what has been widely observed in batch cultures [19], [20], [13], [14], in which a general reduction in hexose consumption, used as carbon source, has been observed. However, this evidence could arise in batch cultures from the combined effects of temperature and growth rate, produced by the reduction in temperature.

The lactate concentration profile (Figure 3b) showed differences in the levels reached, it recorded a 35% increase in lactate production at High-D that Low-D. This difference is also reflected in the increased productivity of lactate at High-D, which is 42% larger than at Low-D (Figure 4b). No differences were observed due to the reduction of the cultivation temperature.

This behavior, resulting from the variation in dilution rate, it has been previously reported in chemostat culture of CHO cells [41] and other cell lines [44], [42], [32]. However, the fact that the specific lactate production were independent of the cultivation temperature differs from the behavior observed in batch cultures [19], [20], [13]. In these cultures, a decrease is observed in the value of the parameter, which is linked to a total reduction of cellular metabolism.

An important indicator of the metabolic state of the cells is the ratio of lactate produced to glucose consumed (Y_Lac/Glc_) [11], [13], [20]. The Y_Lac/Glc_ showed a significant decrease due to decrease in the dilution rate by 42% at 37°C and 34% at 33°C, respectively (Figure 4d). This shows a change in the metabolic fate of the carbon, also indicating a more efficient utilization of glucose at Low-D. These results suggest that the reduction in the Y_Lac/Glc_ in batch culture [8], [20] occurs mainly due to the reduction in the cell growth and is not the result of reducing the culture temperature only.

On the other hand, the consumption profile of glutamate (Figure 3c) showed, how expected, a much lower amino acid utilization compared to glucose [33]. Interestingly, the use of this amino acid (Figure 4c) was significantly affected by the temperature reduction, showing a reduction of 42% independent of the dilution rate used. Similarly, reduction of dilution rate from High-D to Low-D caused a decrease in the q_Glu_ by 52% at 37°C and 66% at 33°C respectively.

The behavior observed, is agree with data in the literature, which indicates a decrease in the consumption of glutamine as an energy source, in CHO cells [41] and other cell lines [44], [42], [32] in chemostat culture as a result of the reduction in the rate of dilution. On the other hand, the effect of mild hypothermia on the consumption of amino acids used as an energy source is controversial. Yoon and colleagues reported in batch culture a reduction in the consumption of glutamine [20] as the main energy source, while others authors in the same culture system, indicate an increase in the amino acid consumption due to the reduction of the temperature of cultivation [13], [8].

## Conclusion

The individual effects of mild hypothermia and specific cell growth rate were evaluated in chemostat cultures at SS. To our knowledge, this is the first report in which these variables have been studied separately. The use of mild hypothermia condition showed a differential effect on the specific productivity of rht-PA, being negative to a condition of High-D (0.017 h^−1^) and negligible to Low-D condition (0.012 h^−1^). However, the decrease of D under the conditions of mild hypothermia, promotes an increase in the specific productivity of protein, suggesting that a condition of mild hypothermia and smaller values of µ promote a greater system productivity. A comprehensive analysis of this behavior shows that the response observed in batch culture corresponds to a combined effect of both variables when using the mild hypothermia condition.

The analysis of the central metabolism revealed that at Low-D, a more efficient metabolism in glucose utilization is presented, lowering the ratio of lactate produced to glucose consumed, with no effect the reduced temperature on this parameter.

These results show that through a suitable experimental approach it is possible to evaluate the differential effect of mild hypothermia and specific growth rate on the production of r-protein in CHO cells, showing that the cellular response in the studied system is primarily modulated by changes in the specific growth rate and to a lesser extent by changes in the culture temperature. Understanding how these variables individually affect the different stages of protein synthesis, is a critical aspect, on which future efforts should be focused.
